# A Hit—A Very Palpable Hit

**Published:** 1859-01

**Authors:** 


"A HIT—A VERY PALPABLE HIT."—Our facetious contemporary, "*The Medical News*," apparently proof against the gloomy influences of the season, has perpetrated a good thing, which, although it is in part at our expense, we cannot refrain from repeating.

"*Mrs. Partington.*—This discriminating lady, we are led to believe, has become a contributor to some of the medical journals; for we find in them several articles which strongly bear the impress of her peculiar and happy style of elucidating subjects.

"Thus—we notice in an editorial article in the *Medical Times and Gazette* for October 30th last, the following statement:—'Bibron is said to be an antidote for the poison of the rattlesnake. M. de Vesey has made experiments to test the correctness of Dr. Hammond's observations, and the results are quite confirmatory of them.'

"A writer in a contemporary in this country seems to have been equally 'exercised' by Bibron's antidote. After giving correctly the formula for its preparation, he remarks: 'As no solvent is mentioned in this formula we are unable to attach any meaning to the dose of ten drops, or to tell its strength.'

"The writer seems not to be aware that bromine is a *liquid*, and we therefore state the fact for his information."

The announcement, that a prescription which is directed to be given in drops is a liquid, must be thankfully received, if not for its novelty, at least for the good nature which prompted its utterance. But our jocose critic "seems not to be aware" that bromine, although a liquid, is a fit substance for a solvent, and that liquid though it be, it is seldom administered medicinally, unless it be in a state of solution. Turner, in speaking of the properties of bromine, says: "It is soluble in water, alcohol, and ether, the latter being the best *solvent*."* If our critic thinks that we have given too great a latitude to the meaning of solution, we would refer him to the chemical definition of the word, as found in Booth & Morfit's "Encyclopedia of Chemistry," which happens to be at our elbow. It reads thus: "The union of a solid, liquid or gas with a liquid in which it disappears or becomes liquid." In illustration, we are told in the same article, "that water dissolves a little ether, and ether a little water," which, if the restricted view of our critic, as to the behavior of a liquid, were sound, ought to be called a very unnatural intimacy and attempt at union between these two liquids, and as such would no doubt cause poor Mrs. Partington to be not a little exercised on the occasion, and to exclaim—"Why should a liquid allow itself to be liquidated, or a liquor-dealer allow himself to be changed into a man in liquor?"

We have said that bromine is seldom administered medicinally, unless it be in a state of solution. In Bell's "Prac-

---

* The italics are ours.

tical Dictionary of Materia Medica," a work to which our "writer," who is so paternally advised by our contemporary, might be supposed to refer, we are told—"An aqueous solution, composed of one part, by weight, of bromine, and forty parts of water, may be prescribed in *doses of five or seven drops*, properly diluted, and flavored with syrup." Very nearly the same language is used in the still more authoritative work of Drs. Wood and Bache, the "Dispensatory of the United States"—"The form in which bromine is employed is aqueous solution," of the same strength as that just described, and in a dose of six drops, several times a day.

With a knowledge of the fact, that bromine is an active irritant poison, one drop of which on the beak of a bird has caused instant death, and that one drop in half an ounce of water produced a sense of heat in the mouth, œsophagus, and stomach, and subsequent colic, our "writer," after repeating the formula for "Bibron's antidote," might very naturally pause and bethink himself of the necessity of a solvent for ten drops of bromine, which was nothing weakened certainly by the addition of iodide of potassium and corrosive sublimate. This dose of ten drops is about sixty-five times the strength of the average dose of six drops of the watery solution just noticed; and hence to reduce the strength of the ten drops to that of the one prescribed in the two works quoted from, would require that the former should find a solvent of more than six drachms and a half of water. The formula entire for "Bibron's antidote" would require to be dissolved in more than a pint and a half of water, to reduce it to the same standard. This *solution* would then be fitted for ordinary extemporaneous prescription in drops, the acrimony of which will be covered by dilution in some mild or agreeable vehicle, syrup, wine, etc. But while we offer good and sufficient reasons for the language held by our "writer," we do not for a moment question the accuracy of these gentlemen, who gave the bromine in such large doses. We would, however, caution our medical brethren against beginning with such doses in cases in which the system is not powerfully impressed by some toxical agent, such as that described by the gentlemen who used Bibron's antidote.

